# Study on short-term clinical observation of the effect of apically repositioned flap combined with free gingival graft to widen keratinized tissue in implant area

**DOI:** 10.4314/ahs.v23i2.38

**Published:** 2023-06

**Authors:** HuanHuan Jiang, LinXiang Liu, Yan Dong, MeiNa Yu, Yue Yuan, Lei Tian

**Affiliations:** Department of Dental Implantology, Wuxi Stomatological Hospital, No.6 Jiankang Road, Liangxi District, Wuxi, China

**Keywords:** Apically repositioned flap, free gingival graft, implant, keratinized tissue

## Abstract

**Objective:**

To analyse the short-term clinical results of the effect of apically repositioned flap combined with free gingival graft to widen keratinized tissue in implant area, so as to provide a basis for its clinical application.

**Methods:**

Fifteen patients with intraoral single or multiple missing teeth, who did not undergo implant restoration or who re-examined after implant restoration completed were included, along with KW less than 1-2 mm on the buccal side of the median line of the alveolar ridge crest in the implant area, or KW less than 1-2 mm on the buccal side of the abutments and dental crown margins. All underwent apically repositioned flap combined with free gingival graft, which were reviewed.

**Results:**

Fifteen patients with missing keratinized gingivae underwent free gingival flap graft, survived with all grafted gingival flaps. Compared with before implantation, significant keratinized tissue widening and area gain were obtained at 1 and 3 months postoperatively.

**Conclusion:**

The free gingival flap graft can significantly widen the buccal keratinized mucosa of the implant, and to some extent maintains the health status of the implant, which is worthy of clinical promotion and application.

## Introduction

Since the discovery of the theory of osseointegration by Professor Branemark in the 1970s, the technique of oral implant placement for fixed and mobile repair of dentition defects and edentulous jaw patients has become a practical and predictable treatment technique with high clinical success rates. However, with the development of implant technology, the definition of criteria for implant success has been gradually transitioned from mere osseointegration success to the attainment of long-term stable functional and aesthetic outcomes 1. It is well known that the absence of teeth will necessarily lead to the resorption of alveolar bone, which will be accompanied by a defect in keratinized tissue. The comprises portions of the free and attached gingiva, both of which extend toward the root side to reach the membrano-gingival junction, that is, the gingival surface from the gingival margin all the way to the membrano-gingival junction is called keratinized tissue. Studies have shown that the dense keratinized tissue structure is conducive to the daily cleaning of implants and can resist various types of mechanical damage, and the loss and thinning of keratinized tissue can lead to the accumulation of plaque around implants and reduced resistance to bacteria, which in turn leads to the occurrence of peri-implantitis 2.

As part of the attached mucosa, keratinized tissue can also resist traction forces generated during mastication and labial movement, limiting the progression of the inflammatory response to the root side^3^. Bassetti et al. 4 defined peri-implant keratinized tissue width < 2 mm as insufficient keratinized tissue, and statistically, up to 37% of implants in the posterior tooth region had insufficient keratinized tissue width < 2 mm buccally. If implant surgery is performed directly without intervention in cases with this partial keratinized tissue defect, complications such as peri-implantitis due to keratinized tissue loss will arise in great numbers in the near future and will not only cause great pain to the patient but also impose a great burden on the clinical work of the physician. Therefore, in cases of keratinized tissue defects in the implant region, incremental reconstruction to keratinized tissue is of great clinical interest to increase the predictability of implant restoration.

Autologous free gingival graft refers to obtaining overlying keratinized supra-epithelial connective tissue from a donor site, which is transplanted into another site in the oral cavity. Free gingival grafts are usually obtained from the palatal mucosa, and because the grafts are derived from autologous tissue, specific connective tissue cells are transferred along with autologous tissue grafts, and after transplantation, most of these cells can be nourished in the plasma circulation during the initial postoperative period as well as in the revascularized blood supply. Therefore, they have a better prognosis 5. Currently, autologous free gingival graft technique is considered the gold standard for soft tissue widening and is the most desirable material for keratinized tissue broadening 6.

The study combined apically repositioned flap and free gingival graft to widen keratinized tissue in implant area for patients with insufficient keratinized tissue (< 2 mm in width). The present investigation is deals to observe the short-term clinical effects of autologous free gingival graft to widen insufficient keratinized tissue in implant areas, providing reference and guidance for its use in clinical applications.

## Material and methods

### Selection and grouping of study objects

The study objects were selected from periodontal patients who underwent implant restoration therapy or post implant review at the Department of Implantology, Wuxi Hospital of Stomatology, from June 2020 to June 2021, and all patients had completed a well-established periodontal system treatment.

### Inclusion criteria

Patients with intraoral single or multiple teeth missing for various reasons who did not undergo implant restoration or who were re-examined after implant restoration was completed, accompanied by the presence of kW less than 2 mm on the buccal side of the median line of the alveolar crest in the implant area, or kW less than 2 mm on the buccal side of the abutments and dental crown margins.

### Exclusion criteria

1. Diabetic patients with poor glycemic control (fasting glucose > 7 mmol / L and glycated hemoglobin > 7.5%; 2. Uncontrolled hypertension (systolic blood pressure > 140 mmHg or diastolic blood pressure > 90 mmHg); 3. Heavy smokers (> 10 cigarettes / D); 4. Patients failed to be reviewed on time for various reasons.

### Procedure

1. **Preparation of the implant bed:** A parallel incision is made parallel to the membrano-gingival joint on the keratinized mucosa at the crests or dental crown margins, the semi-thick flap is turned, the periosteum is preserved, and the membrano-gingival joint is repositioned in the apical direction to form the impacted wound and its size is measured.

2. **Take the strip epithelial free connective tissue:** Take the strip epithelial free connective tissue from the premolars of the hard palate area of the maxilla and the palatal side of the first molar. The surgical area was excluded for inflammation and hyperplasia by first inspection, and then its thickness was detected with periodontal probing. The incision was approximately 2-3 mm from the palatal gingival margin. A vertical incision was made proximally and distally, ranging from a rectangle parallel to the gingival margin. Length and width and the amount required of a seeded bed are comparable. The depth was taken to the submucosa, preserving the periosteum and its overlying connective tissue 1 mm thick, and the cut thickness was approximately 1.5 mm.

3. **To fix the free gingiva of suitable size with epithelium in the recipient area:** Two angular sutures of the proximal gingival ends of free connective tissue were first fixed on the corresponding periosteum, sutured horizontally from the inferior border of free connective tissue to the periosteum, and an “8” word cross sewn on the periosteum of the implanted bed. After suture fixation, the mucosal flap was gently pressed with normal saline moistened gauze for 5 min to exclude the sludging blood beneath so that the graft was snugly placed on the implant bed. The length and width of the free gingival flap used for grafting were recorded.

**Figure 1:**
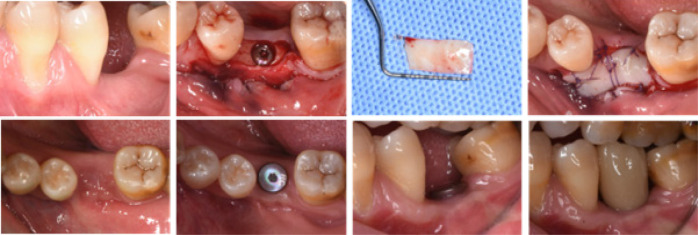
Intraoperative and 1-month and 3-months review photographs of the widened keratinized gingivae

The patient was instructed to rinse the mouth using 0.12% chlorhexidine acetate solution for 2 weeks after surgery, 3 times / d, 1 ~ 2 min / time. Take 5 d cefprozil, 2 times / d, 0.25 g / time postoperatively or change to other antibiotics (roxithromycin or azithromycin) if allergic to cefprozil. Patients may take pain medications (ibuprofen sustained release capsules) based on their own pain. Patients were asked to fill out a visual analogue scale (VAS) questionnaire daily for postoperative pain conditions within 1 week after surgery, and the doses (tablets) of postoperative pain medication were recorded. Sutures were removed 2 weeks postoperatively and questionnaires recollected. The soft tissue healing and KW on the buccal side of the patients were recorded immediately, 1 month, and 3 months after the operation, respectively (Fig. 1).

## Results

Comparison of the width of keratinized tissue on the buccal side of the implant at different time points (Table 1). Compared with the preoperative values, significant keratinized tissue widening was obtained immediately, 1-month and 3-month after surgery, and the keratinized tissue widths were (6.67 ± 1.05) mm, (5.03 ± 1.29) mm, (4.63 ± 1.26) mm, respectively. The width change of keratinized tissue was statistically significantly different one month after surgery compared with the immediate postoperative period (P = 0.001), and there was no statistically significant difference in the width change of keratinized tissue three months after surgery compared with the immediate postoperative period (P > 0.05).

**Table 1:** Comparison of the width of keratinized tissue on the buccal side of the implants at different time points (x ± s)

Grouping	Width (m)
Pre-operative period	0.8±0.38

Immediate postoperative period	6.67±1.05
1 month	5.03±1.29
3 months	4.63±1.26
t_(p)immediate - 1 month_	3.811 (0.001)
t_(p)1 month - 3 months_	0.860(0.397)

Comparison of the keratinized tissue area on the buccal side of the implants at different time points (Table 2). Compared with the preoperative values, significant increases in the keratinized tissue area were obtained immediately, 1 month, and 3 months after surgery, and the keratinized tissue area was (110.97 ± 23.9) mm^2^, (71.73 ± 20.18) mm^2^ and (62.07 ± 16.56) mm^2^, respectively. The area change of keratinized tissue was statistically significantly different one month after surgery compared with the immediate postoperative period (P = 0.001), and there was no statistically significant difference in the area change of keratinized tissue three months after surgery compared with the immediate postoperative period (P > 0.05).

**Table 2:** Comparison of the area of keratinized tissue on the buccal side of the implants at different time points (x ± s)

Grouping	Area (mm^2^)
Preoperative period	12.97±6.44

Immediate postoperative period	110.97±23.9
1 month	71.73±20.18
3 months	62.07±16.56
t_(p)immediate - 1 month_	4.858(<0.001)
t_(p)1 month - 3 months_	1.434(0.163)

The pain condition was analysed at 1 week after operation, and the results were shown in Table 3

**Table 3:** Pain profile 1 week after surgery

Patients	Gender	Age	Tooth position	Pain (points)	Ibuprofen (tablets)
1	F	49	36	3	2
2	M	35	36	2	1
3	M	43	34	1	0
4	M	65	36/37	2	2
5	F	45	42	2	1
6	F	36	46	2	1
7	M	34	24	1	1
8	M	34	35	3	2
9	M	62	36/37	3	3
10	M	34	36/37	3	2
11	F	27	35	2	2
12	M	40	35/36	3	3
13	F	42	46	1	1
14	F	31	35	2	2
15	F	40	36	2	2

**Mean value**		41.13		2.27	1.67

### Statistical analysis

All data of the 15 patients with insufficient keratinized gingival width in this study were processed by SPSS 17.0 software, and the keratinized gingivae width and the free gingival grafted area ratio of the palate were expressed by “x ± s”, using student's t-test, and differences with P < 0.05 were considered statistically significant.

## Discussion

Currently, implant placement has become the modality of choice for the repair of missing teeth. But a reduced or absent keratinized gingiva in the edentulous area is a frequently encountered condition in the clinic. In 1972, Lang 7 considered that at least 2 mm wide keratinized gingivae (where 1 mm is the attached gingiva) was necessary for periodontal health after conducting studies on natural teeth, and since then, there has been a debate about the significance of width of keratinized mucosa for natural periodontal health and peri-implant health. Some scholars, such as Maynard 8 and Stetler 9, support the view of Lang that to maintain periodontal health, there should be at least 2 mm wide keratinized mucosa on the buccal side of the tooth, and on the contrary, there are also many scholars who believe that the width size of keratinized mucosa on the buccal side has no significant effect on natural periodontal health 10, 11. In the 1990s, Berglundh et al., 12 and Lindhe et al. 13 et al. showed both similarities and differences between the architecture of the implant and the native peri-odontium, the latter of which makes the implant more vulnerable to damage from the external environment relative to the native tooth. Considering the requirements for long-term implant stability, an increasing number of studies have shown that for implants, a buccal keratinized mucosa width of ≥ 2 mm helps maintain peri-implant tissue health 14-18.

As early as the 1960s to 1970s, the use of free gingival graft combined with apically repositioned flap has been used by physicians to improve the peri-implant keratinized gingivae width to maintain long-term implant stability and health. It has been demonstrated that grafts derived from the palatal mucosa can maintain tissue specificity to produce keratinized tissue. Therefore, the palatal autologous free gingival graft technique is considered the gold standard for soft tissue augmentation and is the most desirable material for keratinized tissue widening 5, which offers the advantages of stable treatment outcomes and great predictability.

Through this clinical observation, apically repositioned flap combined with free connective tissue graft is beneficial to the increase of keratinized gingivae width, on the other hand, it also has a certain effect on increasing the thickness of soft tissue in the implant area. Similar to the conclusions of previous explorations, Thoma 19 et al., based on a systematic summary of the efficacy of various procedures for peri-implant soft tissue augmentation, used twenty publications to explore the Meta-analysis and concluded that simple keratinized gingivae graft, apically repositioned flap, and connective tissue graft are all able to widen the keratinized gingivae by 1.4-3.3 mm. After a comparison of these three types of grafts, it was found that the keratinized gingivae increment was not much after a single application of the apically repositioned flap, but the patient's postoperative discomfort and length of operation were greatly reduced. Regarding keratinized tissue increments of free gingival graft, Wang Qi 20 studied that the keratinized tissue increments of (3.44 ± 1.64) mm could be obtained in the free gingival graft group at 3 months postoperatively, and Basegmez et al. 21 showed that the attached mucosa increments of 2.36 mm could be obtained at 1 year postoperatively. It has been showed that keratinized tissue increments of 3.0-3.3 mm can be obtained 6 months after free gingival graft, which is similar to the results of this study. Schmitt et al. 22 showed that the keratinized tissue increments of about 8.69 mm could be obtained in the group with free gingival graft at 3 months postoperatively, and about 8.07 mm could be obtained in the group with collagen matrix, which were both significantly higher than those in the present study, and the keratinized tissue increments were determined by intraoperative grafted tissue width and the shrinkage of grafted tissue postoperatively. Differences in the surgical position, clinical procedure of the operating personnel, and timing of the procedure, soft tissue biotype, and human race may contribute to the differences in outcomes observed indifferent studies. Wang Qi 20 observed that, one month after surgery, the graft flap area will decrease, and by the third month there will still be some shrinkage, but the amount of shrinkage will decrease gradually and then stabilize, a large amount of shrinkage occurs in the graft tissue 2 weeks after surgery. The shrinkage of keratinized tissue in the free gingival graft group is 34% at 3 months after surgery, a short-term clinical study by Schmitt et al. 22, on the effect of collagen matrix and free gingival graft to widen peri-implant keratinized tissue showed that there was significant shrinkage of keratinized tissue in both groups within 1 month postoperatively, with 32.98% and 28.35% shrinkage of keratinized tissue between the collagen matrix group and the free gingival graft group at 3 months postoperatively. Sanz et al. 23 showed that keratinized tissue had a shrinkage of up to 67.2% within 1 month after surgery and that about 8% of the grafted tissue still had shrinkage between 1 month and half a year after surgery. In addition, differences in timing of surgery, thickness of grafted tissue, soft tissue biotype, and human race may contribute to differences in shrinkage across studies. Larger sample sizes and longer observation periods should be provided for the follow-up studies, so as to conduct further studies on the above factors.

An unfavourable aspect of autologous free gingival graft has been reported to be the increased postoperative pain of patients due to the need to open the second surgical area as well as the healing on the palatal side 24-27, The study by Giovanni Zucchelli 28 showed that patients with free gingival graft did not experience increased postoperative pain, discomfort, and bleeding compared with patients with subepithelial connective tissue graft, and there was no statistically significant difference in analgesic consumption. Data from the current study suggest that the depth of tissue removal rather than the type of palatal wound healing (primary vs. secondary) influences postoperative analgesic consumption. In the studies reported in the literature, the more painful course during the postoperative course in patients undergoing FGG surgery may be due to the thicker nature of the grafts and the greater depth of palatal soft tissue that is reached during the harvest technique, and is not (or not only) due to the different types (primary or secondary) of palatal wound healing. Our study also found that the pain medication usage of patients after free gingival graft was not large and the degree of pain was also basically within an acceptable range. Therefore, as a better technique to widen the peri-implant keratinized gingivae, its postoperative pain is acceptable to patients.

Keratinization tissue widening procedures are able to improve peri-implant plaque mastery and promote peri-implant tissue health at implant sites where keratinized mucosa is absent or insufficient (< 2 mm). So, this surgery is advocated, present-day findings suggest that apically repositioned flap combined with free gingival graft is one of the effective means to improve the predictability of width of peri-implant keratinized mucosa. It can get more keratinized tissue and good stability for long time. However, the free gingival graft inherits the initial appearance of the soft tissues of the palate and does not match the color of the surrounding tissues. Some data prove that there are good incremental KTW around implants as well as alternative materials and biologics, but it is also necessary to go through a sufficiently large number of rigorously designed randomized clinical trials with longer follow-up periods to clarify the effectiveness and advantages of each surgical approach and alternative materials. However, it was always unsatisfactory in terms of aesthetics. There is also the fact that the application of autologous soft tissue grafts has to look for another surgical area in the palate, which necessarily aggravates the postoperative pain of the patients, the palate has blood vessels and nerves, and the amount of autologous soft tissue that can be obtained is still insufficient. Utilizing apically repositioned flap combined with free gingival graft to treat the insufficient amount of peri-implant keratinized tissue in medical implant is still the best choice of keratinized tissue widening operation until now. After change of times, the material capable of obtaining autologous tissue grafts emeres in endlessly, among them are the advent of new materials such as AOM and XCM, which are able to greatly improve the aesthetics of the postoperative area, relieve patients' postoperative pain, improve the sensitivity of the surgical procedure and have a lower medical cost. However, although xenogeneic tissues of alternative materials have some advantages, the problem of large tissue shrinkage is not solved, and how long its stable properties are maintained remains to be examined. In the future period, the development of novel autograft substitutes will be the direction of this industry.

## Conclusion

The free gingival flap graft can significantly widen the buccal keratinized mucosa of the implant, and to some extent maintains the health status of the implant, which is worthy of clinical promotion and application.
